# Neural responses to emotional expression information in high- and low-spatial frequency in autism: evidence for a cortical dysfunction

**DOI:** 10.3389/fnhum.2014.00189

**Published:** 2014-04-09

**Authors:** Corrado Corradi-Dell'Acqua, Sophie Schwartz, Emilie Meaux, Bénedicte Hubert, Patrik Vuilleumier, Christine Deruelle

**Affiliations:** ^1^Swiss Center for Affective Sciences, University of GenevaGeneva, Switzerland; ^2^Laboratory for Neurology and Imaging of Cognition, Department of Neuroscience and Clinic of Neurology, University Medical CenterGeneva, Switzerland; ^3^Hôpital Rivière-de-Praires, University of MontréalMontréal, QC, Canada; ^4^CNRS, Institut de Neurosciences de la Timone, Aix-Marseille UniversitéMarseille, France

**Keywords:** autism, facial expression, emotion expression, spatial frequency, fMRI

## Abstract

Despite an overall consensus that Autism Spectrum Disorder (ASD) entails atypical processing of human faces and emotional expressions, the role of neural structures involved in early facial processing remains unresolved. An influential model for the neurotypical brain suggests that face processing in the fusiform gyrus and the amygdala is based on both high-spatial frequency (HSF) information carried by a parvocellular pathway, and low-spatial frequency (LSF) information separately conveyed by a magnocellular pathway. Here, we tested the fusiform gyrus and amygdala sensitivity to emotional face information conveyed by these distinct pathways in ASD individuals (and matched Controls). During functional Magnetical Resonance Imaging (fMRI), participants reported the apparent gender of hybrid face stimuli, made by merging two different faces (one in LSF and the other in HSF), out of which one displayed an emotional expression (fearful or happy) and the other was neutral. Controls exhibited increased fusiform activity to hybrid faces with an emotional expression (relative to hybrids composed only with neutral faces), regardless of whether this was conveyed by LSFs or HSFs in hybrid stimuli. ASD individuals showed intact fusiform response to LSF, but not HSF, expressions. Furthermore, the amygdala (and the ventral occipital cortex) was more sensitive to HSF than LSF expressions in Controls, but exhibited an opposite preference in ASD. Our data suggest spared LSF face processing in ASD, while cortical analysis of HSF expression cues appears affected. These findings converge with recent accounts suggesting that ASD might be characterized by a difficulty in integrating multiple local information and cause global processing troubles unexplained by losses in low spatial frequency inputs.

## Introduction

Autism Spectrum Disorder (ASD) is a pervasive neurodevelopmental disorder characterized by dysfunctional socialization and communication, with the emergence of stereotyped and repeated behavior. Although this disorder is mostly known for its social symptoms, a wealth of studies converge in reporting atypicalities in elementary aspects of perception, as in the case of visual processing of (emotional and neutral) facial expressions (see Harms et al., 2010; Gaigg, 2012; Weigelt et al., 2012, for meta-analyses and reviews).

A wealth of studies based on abstract and geometrical stimuli, suggest that in ASD individuals have difficulties in processing visual stimuli in a global fashion, focusing instead on details and local information (e.g., Dakin and Frith, 2005; Happé and Frith, 2006; Mottron et al., 2006). These accounts can potentially explain also ASD atypical processing of faces, especially considering that facial properties (identity, gender, emotional expressions, etc.) are not usually processed by the analysis of isolated local features, but of how all different features relate one another at the global level (configural processing). In this perspective, tasks asking neurotypical individuals to assess the sameness of two faces usually report poorer performance when the standard spatial relation between the parts is distorted, as for upside-down faces (Valentine, 1988), composites made of two aligned half-faces from different people (Young et al., 1987), or faces with scrambled parts (Tanaka and Farah, 1993). However, studies implementing the same tasks in individuals with ASD have reported mixed findings with some describing them as not influenced (Van Der Geest et al., 2002; Joseph and Tanaka, 2003; Teunisse and de Gelder, 2003; Rondan and Deruelle, 2004; Riby et al., 2009) or less influenced than Controls (Hobson et al., 1988; López et al., 2004; Barton et al., 2007; Pellicano et al., 2007, see also Weigelt et al., 2012), but others describing equal effects as in neurotypical individuals (Teunisse and de Gelder, 2003; Rouse et al., 2004; Lahaie et al., 2006; Gross, 2008). Such variability could reflect the important heterogeneity of the ASD population, in which diagnostic symptoms are expressed differently across individuals, maybe confounded by age or attentional factors (Rondan and Deruelle, 2007), and/or possibly stem from the development of compensatory neuronal mechanisms (Gaigg, 2012; Dickstein et al., 2013).

**Figure 1:**
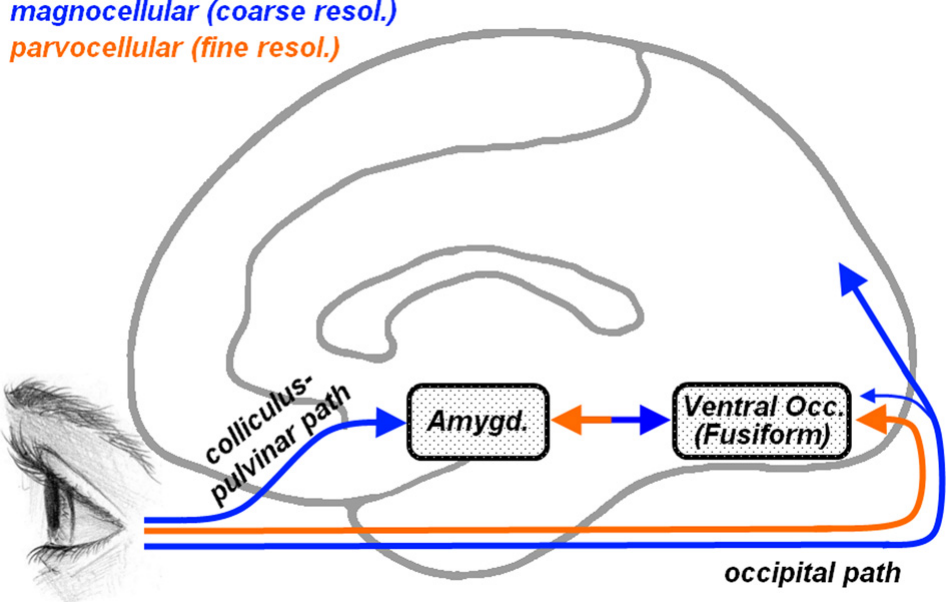
**Representation of the cortical and subcortical pathways for visual inputs overlaid on a schematic human brain (medial view)**. Orange arrows describe parvocellular pathways conveying fine (high-frequency) visual information, whereas blue arrows describe magnocellular pathways conveying coarse (low-frequency) information. Parvocellular inputs reach the striate cortex and project to ventral occipital regions, including the fusiform gyrus. Magnocellular inputs project more dorsally toward the parietal cortex and, to a lesser extent, also toward the ventral occipital cortex. Another magnocellular pathway reaches the amygdala via a subcortical *colliculus-pulvinar* projection. Fusiform and amygdala are reciprocally connected.

To better characterize the face processing atypicalites observed in ASD, several studies have focused on the spatial frequency at which specific information is conveyed, suggesting that distinct frequencies might play different roles in face processing (Deruelle et al., 2004, 2008; Rondan and Deruelle, 2004; Boeschoten et al., 2007a; Vlamings et al., 2010). Indeed, local information can be processed only through high-spatial frequencies (HSF), whereas global configurations can be retained also from low spatial frequencies (LSF). It is well known that HSF visual information is carried by parvocellular pathways (see Figure 1, orange arrow) which reach the striate cortex and project almost exclusively to ventral occipito-temporal structures, including that part of the fusiform cortex which processes face stimuli (Fusiform Face Area [FFA], Kanwisher et al., 1997). LSF information instead is conveyed by magnocellular pathways (Figure 1, blue arrow) which project mostly to dorsal to parietal regions and, in less extent, to ventral cortical visual areas (Livingstone and Hubel, 1987, 1988). In addition, however, it has been proposed that the amygdala, a medial temporal structure critically involved in processing emotional expression in faces (Vuilleumier and Pourtois, 2007; Pessoa and Adolphs, 2010), may receive direct subcortical inputs from an additional collicular-pulvinar projection of magnocellular pathways (De Gelder et al., 1999; Morris et al., 1999), allowing the amygdala and ventral visual stream to receive coarse (LSF), but fast, information about facial emotional expressions (Vuilleumier et al., 2003, 2004; Winston et al., 2003b; Carretié et al., 2007; Vuilleumier and Pourtois, 2007). In this perspective, the frequent reports of atypical fusiform and/or amygdala responses to face stimuli in ASD (Baron-Cohen et al., 2000; Critchley et al., 2000; Schultz et al., 2000; Pierce et al., 2001; Hall et al., 2003; Hubl et al., 2003; Wang et al., 2004; Grelotti et al., 2005; Ashwin et al., 2007; Kleinhans et al., 2008; Scherf et al., 2010) raise the question of whether these effects might depend on differential visual frequency information conveyed by parvocellular (cortical) or magnocellular (also subcortical) pathways.

A number of studies have employed electrophysiological recording or behavioral techniques in children with ASD using high or low spatial filtered stimuli. While some results suggested that ASD affects preferentially the visual pathway conveying LSF (Deruelle et al., 2004, 2008; Boeschoten et al., 2007a; Vlamings et al., 2010), similar approaches in adults reported an ability to process LSF expressions comparable to that of neurotypical individuals (Rondan and Deruelle, 2004). To the best of our knowledge, no study has ever tested directly how, in ASD, the fusiform gyrus and the amygdala respond to HSF and LSF information in human faces.

**Figure 2:**
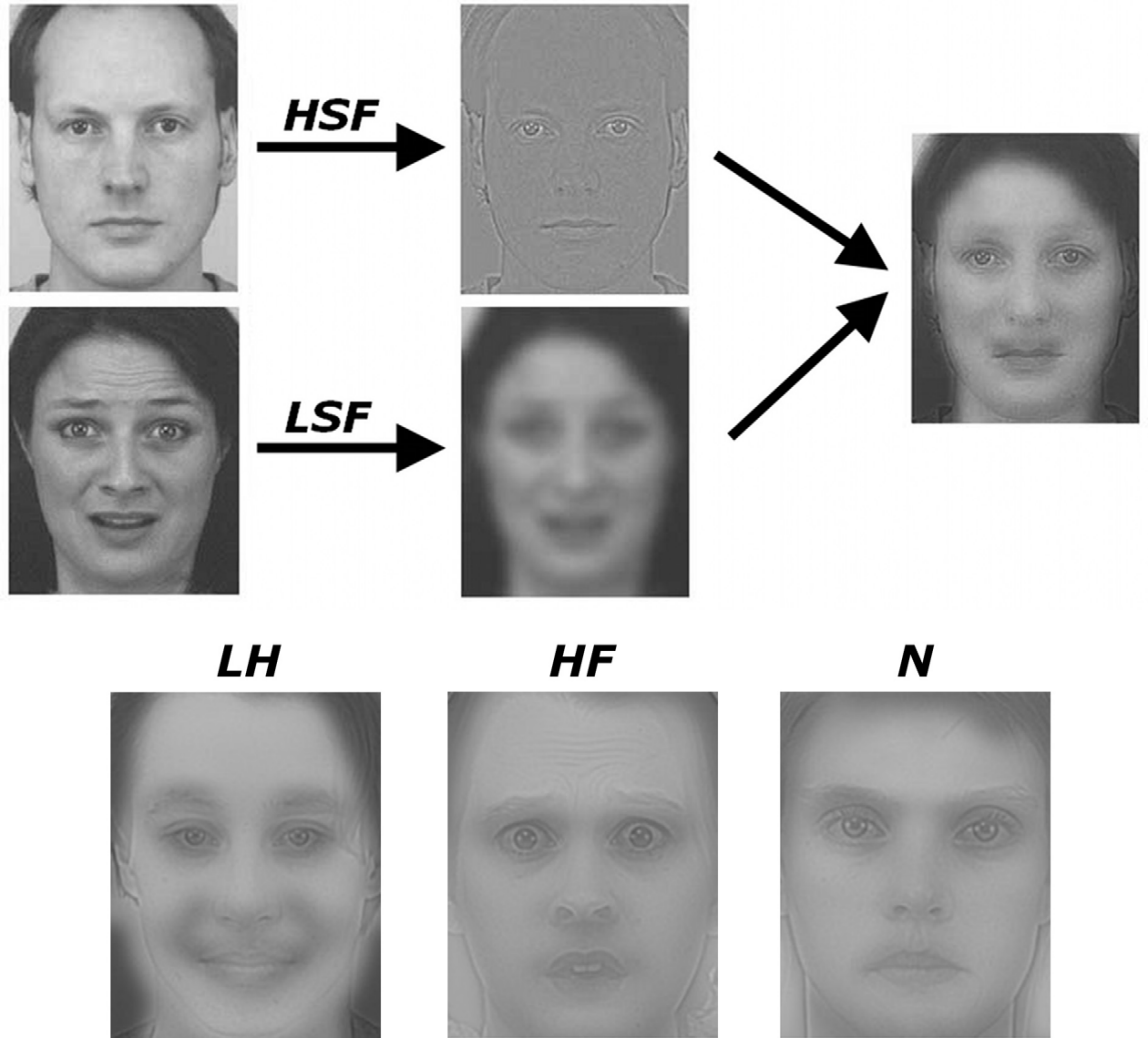
**Schematic representation of how the hybrid stimuli were created**. Male and female faces were selected from a validated database and subjected to spatial frequency filtering. Hybrid faces were then created by overlaying a high-spatial frequency (HSF) face with a low-spatial frequency (LSF) face of opposite gender. Emotional expressions (in either the HSF or LSF face) were always overlaid with a neutral facial expression of opposite gender and opposite filtering. Three stimuli examples are also displayed on the lower part of the Figure. A HSF neutral face overlaid with a happy LSF face (condition LH), a HSF fearful face overlaid with a neutral LSF face (condition HF) and a control condition containing neutral faces in both HSF and LSF bands (N). Full details in the text.

In the present study, we showed to adults with ASD and matched Controls hybrid facial stimuli, which were generated according to a methodology used in previous studies (Schyns and Oliva, 1999; Winston et al., 2003b), by merging a HSF face with a LSF face of opposite gender (see Figure 2). These stimuli are particularly suited for our purpose as they offer to the observer both high- and low- extremes of the frequency spectrum of faces in the same stimulus, allowing us to determine the band in which specific information is preferentially selected for face processing and responded to in different brain areas. Critically, in addition to mixing opposite genders in each spatial frequency band, our hybrid stimuli were made of the combination of a neutral expression and an emotional expression, with the latter being either fearful or happy and contained either in the HSFs or LSFs (counterbalanced across the gender dimension). In order to probe for differential responses to emotion expressions triggered by one or the other frequency bands, we engaged our participants in a gender discrimination task in which they had to report the apparent gender of each face. In a separate condition, our participants instead had to watch passively each stimulus, allowing us to determine any influence of different task demands. This led to a factorial design with group (ASD participants, Controls), frequency (emotional expressions conveyed by HSF, LSF), valence (fearful, happy expressions), and task (gender discrimination, passive viewing). For high-level baseline, we used (in each task) hybrid stimuli with no emotional expression (i.e., mix of neutral female and neutral male). Following previous studies on neurotypical individuals, we expected increased activity in ventral visual cortex (including the fusiform gyrus) when Controls discriminated faces in which emotions were conveyed by either HSFs or LSFs (as opposed to neutral expressions), as evidence of parvocellular cortical and magnocellular visual inputs respectively (Vuilleumier et al., 2001, 2003; Winston et al., 2003b; Rotshtein et al., 2007). The critical question, however, was whether these parvo- and magnocellular neural signatures were observable also in individuals with ASD. We reasoned that if ASD affects the direct subcortical inputs to the amygdala (Vuilleumier et al., 2003, 2004; Carretié et al., 2007; Rotshtein et al., 2007), ASD individuals should exhibit a reduced neural response to emotional expressions conveyed by LSFs. On the other hand, if ASD individuals present a reduced neural response to emotions mediated by HSFs, this can be interpreted exclusively as reflective of an effect to the cortical path.

## Materials and methods

### Participants

Two groups were included in the experiment. The first group comprehended 13 high-functioning adults males with autistic spectrum disorder (ASD) recruited from the database of the Specialized Clinic for Pervasive Developmental Disorders of Rivière-des-Prairies Hospital. Diagnosis of autism was established with the Autism Diagnostic Interview-Revised (ADI-R; Lord et al., 1994) and validated by a standardized assessment with the Autism Diagnosis Observation Schedule (ADOS-G, module 3 or 4; Lord et al., 2000). All participants from the clinical group met the diagnostic criteria for autism or Asperger syndrome according to both instruments. The second group comprehended 15 matched male participants with typical development recruited from the same database.

Some of the participants were excluded from the overall analysis due to technical problems occurred during the acquisition phase and due to head-movement artifacts in the BOLD signal. Therefore, the overall analysis was run on two homogeneous groups of 10 individuals each. Participants from both groups completed one of the Wechsler Intelligence scales (WAIS-R, WAIS-III) and the Edinburgh questionnaire (Oldfield, 1971). ASD and Control participants were group-wise matched according to their IQ [Manova on all IQ variables: Pillai's trace = 0.15, *F*_(3,15)_ = 0.92, n.s.], age and handedness. Table 1 summarizes the participants' demographic characteristics. Each participant gave informed consent to participate in the study and received monetary compensation. The study was formally approved by the ethics committee of Rivière-des-Prairies Hospital and the committee for ethics and research of Regroupement Neuroimagerie/Québec (CMER-RNQ). All participants were naïve to the purpose of the task.

**Table 1:** **Demographic and clinical characteristics of the sample**.

	**ASD participants (*n* = 10)**	**Neurotypical controls (*n* = 10)**
Age (years)	21.50 [19.50, 23.50]	21.55 [19.89, 24.84]
Full scale IQ	100.90 [94.80, 110.60]	109.50 [104.90, 114.90]
Performance IQ	98.70 [92.90, 104.80]	103.00 [99.33, 109.70]
Verbal IQ	102.50 [96.20, 112.90]	111.78 [105.22, 117.11]
Diagnosis	6/10 Asperger syndrome	
	4/10 High-functioning autism	

*Average age and IQ values are displayed in average values with bootstrap estimated 95% confidence intervals. None of the displayed measures varied significantly across groups with α = 0.05*.

### Stimuli

Our experimental stimuli were hybrid images built by combining a face composed only by HSFs with a face composed only by LSFs of opposite gender, whose expression was also separately manipulated (see Figure 2). The detailed procedure was adapted from the one used by previous studies (Schyns and Oliva, 1999; Winston et al., 2003b) and can be summarized as follows. We took pictures of emotional (happy and fearful) and neutral facial expression, displayed from a frontal point-of-view, from the Karolinska Directed Emotional Faces database (A series). Validation studies on stimuli from this database (Goeleven et al., 2008) confirmed that happy and fearful expressions were matched for intensity and arousal, and both associated with higher recognition scores than neutral expressions. These images were desaturated, scaled to a size of about 5.30° (horizontal) × 6.80° (vertical) of visual angle and, subsequently, filtered in Fourier space, using a Butterworth filter to remove either high spatial frequencies (above 24 cycles/face [c/fw], corresponding to about 4 cycles/degree of visual angle [c/deg]) or low spatial frequencies (below 6 c/fw, corresponding to about 1 c/deg). Hybrid stimuli were then created by overlapping one HSF face and one LSF face into a single stimulus (see Figure 2). The eyes and mouth position was matched between the LSF and HSF images in order to obtain a visual overlap yielding the percept of single face. Critically, faces in the LSF and HSF images were chosen so that they always had an opposite gender (one female, one male) and could display different emotional expressions. This manipulation led to the following five conditions of interest: high fearful stimuli, composed by a fearful HSF face and a neutral LSF face (HF); high happy stimuli, composed by a happy HSF face and a neutral LSF face (HH); low fearful stimuli, composed by a neutral HSF face and a fearful LSF face (LF); low happy stimuli, composed by a neutral HSF face and a fearful LSF face (LH); neutral stimuli (high-level control), composed by a neutral HSF face and a neutral LSF face (N). For each of these conditions we built 32 different images, each of which was presented twice during the experimental session (32 images × 2 repetitions × 5 conditions = 320 face stimuli). In half of these 32 pictures of each emotional condition, the emotional expression was conveyed by the female face, whereas in the remaining half the emotional expression was conveyed by a male face.

### Experimental setup

The 320 face stimuli used in the study were presented to participants in an event-related fashion. On each experimental trial, one hybrid face was shown to the participant for 83 ms and followed by an inter-stimulus interval of variable duration (range 2500–12500 ms) in order to improve sensitivity of fMRI BOLD measurements. To encourage participants to keep their gaze on the center of the screen, a fixation cross was present during the inter-stimulus interval. The whole experiment was organized into four experimental sessions, each comprehending 80 trials (16 per condition) and lasting about 4 min. Among these four sessions, two were associated with an active gender detection task: since participants were not aware that the hybrid stimuli were created by faces of opposite genders, they were requested to indicate, as fast as possible, its apparent gender by pressing one of two possible keys with either hand (e.g., left hand for male response, right for female, counterbalanced across participants). Previous studies using hybrid stimuli filtered at the same cut-offs found that, with such short stimulus presentations, participants rely with comparable likelihood on LSF and HSF information to make their gender judgments, as they report the gender of the LSF face on ~50% of the trials (Schyns and Oliva, 1999; see also Winston et al., 2003b, in which the LSF face was chosen 60% of the trials). The two remaining sessions had no active task, and participants were simply requested to pay attention to each and every face. The order between passive and task-positive sessions was counterbalanced across subjects.

The stimuli were presented using E-Prime 1.0 (Psychology Software Tools, Inc.) and projected inside the scanner bore with a LCD projector on a screen subtending about 19° (horizontal) × 14° (vertical) of visual angle. Key-presses were recorded on an MRI-compatible bimanual response button box. Participants were instructed to press one of two possible keys, placed at each hand's reach, to indicate their responses.

### Face functional localizer

Our study aimed at investigating the sensitivity to band-filtered face information in key areas of the core face processing system, particularly fusiform cortex and amygdala (Haxby et al., 2000; Gschwind et al., 2012). To this aim, we mapped the face processing network in both groups with an unbiased (not band-filtered) set of face stimuli. We therefore carried out an independent scanning session adapted from previous studies (Schwarzlose et al., 2005; Spiridon et al., 2006) and structured as follows. Participants were presented four blocks of gray-scale full-band face photographs alternating with four blocks of gray-scale house photographs. Photos were displayed centrally, and had a size of about 9.82° × 9.82°). Within each block, there were 18 face/house specimens each presented for 750 ms followed by an interstimulus interval of 500 ms. Each block lasted of about 22 s each and was immediately followed by another. Whilst perceiving these images participants performed a 1-back task, in which they had to signal through key-press whether the picture in the current trial was identical to the one in the previous trial. The experiment was built so that a positive response from the participant was expected only in two trials in each block. The whole localizer session lasted about 3 min and always followed the four main experimental sessions.

### Imaging processing

#### Data acquisition

The study was conducted in the neurofunctional imagery unit at the research center of the geriatric institute of Montreal. A Siemens Trio 3-T whole-body scanner was used to acquire gradient-echo planar T2-weighted MRI images with blood oxygenation level dependent (BOLD) contrast. The scanning sequence was a trajectory-based reconstruction sequence with repetition time (TR) of 2160 ms, echo time (TE) of 30 ms, flip angle of 90°, in-plane resolution 64 × 64, 36 slices, slice thickness of 3 mm, and no gap between slices. A structural image was of each participant was also recorded with a T1-weighted MPRAGE sequence (176 slices, *TR* = 9.7 msec, *TE* = 4 ms, flip angle = 12°, in-plane resolution = 256 × 256, 1 × 1 × 1 mm voxel size).

#### Preprocessing

Statistical analysis was performed using the SPM software (http://www.fil.ion.ucl.ac.uk/spm/). For each subject, all functional images were realigned, slice-time corrected to allow a whole volume to be treated as a single data point, normalized to a template based on 152 brains from the Montreal Neurological Institute (MNI), resliced at a voxel size of 3 × 3 × 3 mm, and then smoothed by convolution with a 8 mm full-width at half-maximum (FWHM) Gaussian kernel.

#### First-level analysis

Data from each participant were analyzed using the General Linear Model (GLM) framework implemented in SPM. For the face localizer session, we modeled each of the two active conditions (faces, houses) with a boxcar function. For the main experimental sessions, the trial onsets from each condition of our design were modeled with a delta (stick) function. Critically, whereas in the two passive viewing sessions we modeled only the main five conditions of our design (HF, HH, LF, LH, N), in the gender discrimination task we also took into account participants' response on every trial (see Winston et al., 2003b). Thus, for each of the five main conditions, we modeled separately those trials in which participants made their gender judgments on the basis on visual cues conveyed by LSFs (e.g., HFL, HHL, LFL, LHL, NL), those trials in which participants judged gender based on HSFs (HFH, HHH, LFH, LHH, NH), and also those few trials in which responses were omitted (if any). Each regressor was convolved with a canonical hemodynamic response function as implemented in SPM. To account for movement-related variance, we included, for each session, six differential movement parameters [x, y, and z translations (in millimeters) and pitch, roll, and yaw rotations (radiants)] as covariates of no interest. Low-frequency signal drifts were filtered using a cutoff period of 128 s.

#### Second level analysis

For the functional localizer, we calculated for each participant the contrast describing the differential activity *Faces > Houses*. These contrasts were fed in a second-level independent sample *t*-test, under the assumption of unequal variance between the groups. This test allowed us to investigate both effects of *Faces* vs. *Houses* in Controls and ASD participants, as well as cross-over interaction effects.

For the main experiment, we considered for each subject 15 contrast images. 10 of them were computed from the gender discrimination task, and concerned activity associated with the five main conditions and the two possible responses (i.e., HFL, HFH, HHL, HHH, LFL, LFH, LHL, LHH, NL, NH). The remaining five concerned activity in the five conditions of interest (i.e., LF, LH, HF, HH, N) during the passive viewing sessions. These contrasts were fed into second-level flexible factorial design with “conditions” as a within-subject factor, “group” as between-subject factor and “subject” as random factor, using a random effects analysis (Penny and Holmes, 2004). In modeling the variance components, we allowed each of these three factors to have unequal variance between their levels. Activations in these analyses were considered as significant if exceeding an extent threshold allowing *p* < 0.05 correction for multiple comparison for the whole brain (corresponding to 59 and 63 consecutive voxels, for the localizer and main experiment respectively—Friston et al., 1993), with an underlying height threshold corresponding to *p* < 0.001 uncorrected [*t*_(18)_ > 3.61 and *t*_(250)_ > 3.13, for the localizer and main experiment].

## Results

### Behavioral results

To obtain a measure of spatial frequency biases in face processing for different conditions in each group, we analyzed the rate at which participants selected the gender of the LSF face in the hybrid stimuli. In this measure, values greater than 0.5 reflect experimental conditions in which participants relied more on the LSF information to make gender judgments, whereas values smaller than 0.5 reflect conditions in which participants relied more on the HSF information.

**Figure 3:**
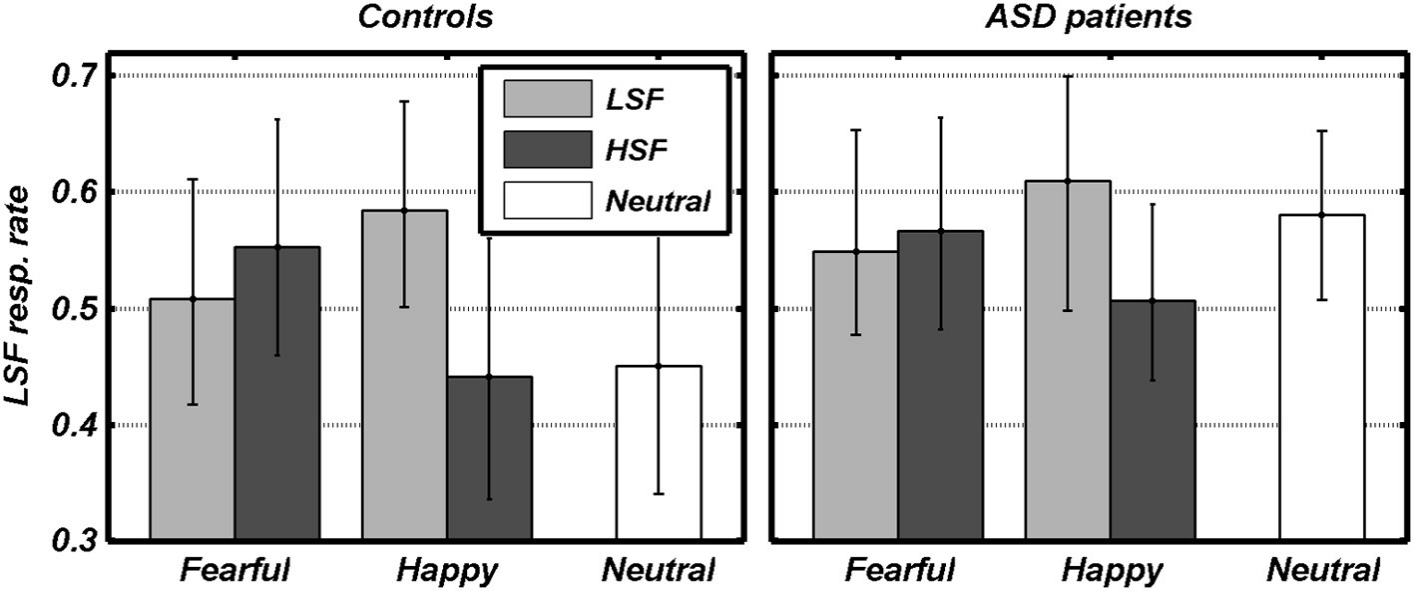
**Behavioral data**. The rate of gender judgments made according to the LSF information in each condition is plotted against the valence of facial expressions. Dark and light gray bars refer to the different spatial frequencies conveying emotional expression. White bars refer to control trials with no emotional expression. Data from each group are displayed in separate subplots. Error bars refer to bootstrap-based 95% confidence intervals of the mean.

We first analyzed the conditions in which an emotional expression was displayed through a 2 × 2 × 2 Repeated measures ANOVA, with the FREQUENCY containing the emotional expression (HSF, LSF) and the VALENCE of this emotional expression (Fearful, Angry) as within-subject factors, plus participant GROUP (ASD individuals, Controls) as between-subjects factor. We found a significant main effect of FREQUENCY [*F*_(1, 18)_ = 9.79, *p* < 0.01], reflecting that overall participants relied more on LSF information [average 0.56, bootstrap-estimated 95% confidence intervals of the average (0.46, 0.64)], rather than on HSF [0.52 (0.40, 0.62)]. However, this LSF-bias also depended on the valence of the emotion expression (see Figure 3). Thus, whereas the VALENCE main effect was not significant [*F*_(1, 18)_ = 0.14], this factor interacted significantly with FREQUENCY [*F*_(1, 18)_ = 18.48, *p* < 0.001]. Figure 2 shows that, in both groups, gender judgments were more LSF-biased when low frequencies conveyed happy expressions, as opposed to fearful [*LH* > *LF*: *t*_(19)_ = 2.39, *p* < 0.05]. Instead, judgments were more HSF-biased when high frequencies conveyed happy, as opposed to fearful, expressions [*HF* > *HH*: *t*_(19)_ = 2.96, *p* < 0.01]. The factor GROUP yielded no significant main effect nor interaction [*F*s_(1, 18)_ < 1.00]. Visual inspection of Figure 3 suggests that ASD individuals might be more LSF-biased than controls, although no significant effect of the factor GROUP was found. However, this initial analysis did not comprehend the high-level control condition in which neutral facial expressions were presented. We therefore also tested for putative group differences in LSF-rate, both when taking each of the five main conditions (thus including N) separately, and when averaging them together. None of these tests led to a significant effect [|*t*|_(18)_ always < 1.60].

Furthermore, for each condition, we computed the median time [in milliseconds (ms)] necessary to deliver a response (Response Times) and analyzed it in a similar fashion as above. In this analysis we also tested for any putative effect of the participants' choice. We therefore analyzed the emotional conditions in a 2 × 2 × 2 × 2 repeated measures ANOVA with FREQUENCY (HSF vs. LSF), VALENCE (Fear vs. Happy), and CHOICE (HSF vs. LSF gender) as within-subject factor and GROUP (Controls vs. ASD individuals) as between subject factor. We found a significant main effect of FREQUENCY [*F*_(1, 18)_ = 4.96, *p* < 0.05], reflecting faster responses when the emotional expression was conveyed by LSF [825.50 ms (735.41, 908.49)], as opposed to HSF [870.51 (769.19, 944.95)]. No other main/interaction effect, including those associated with the factor CHOICE, was significant [*Fs*_(1, 18)_ < 4.27, *p*s > 0.05].

### Neural responses

**Figure 4:**
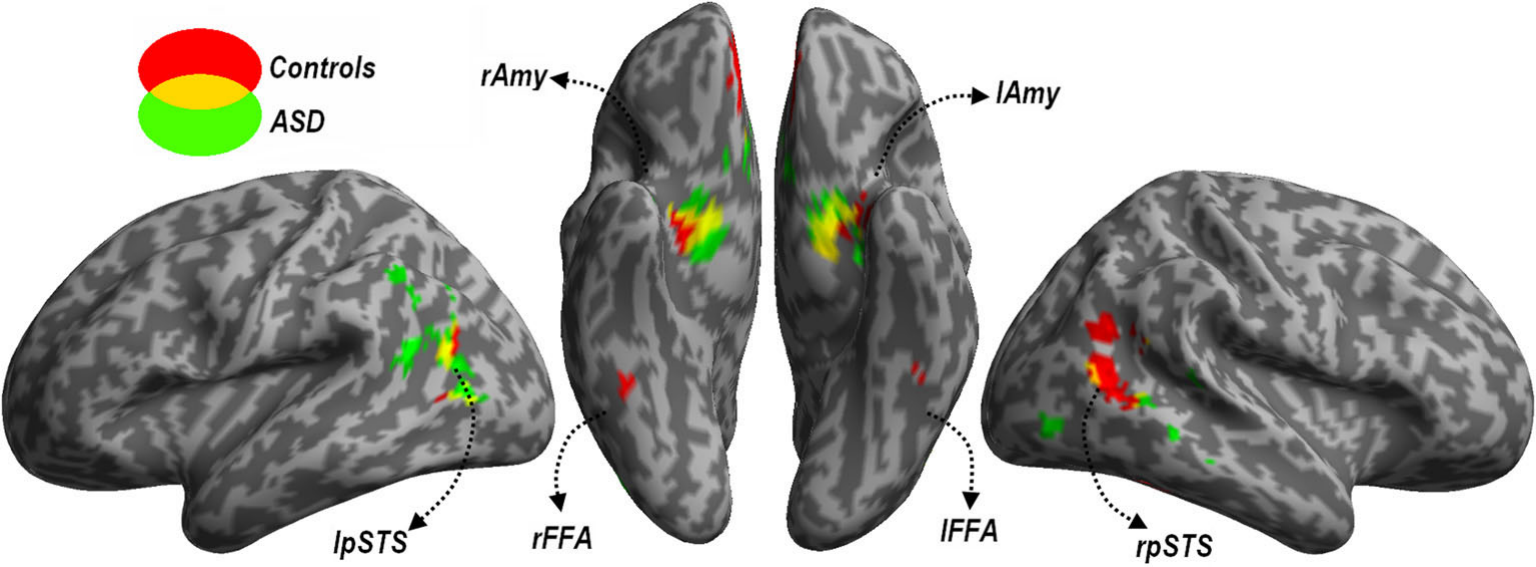
**Localizer session**. Whole-brain maps showing significant increase of neural activity associated with the perception of *Faces > Houses* from the localizer session. Data from neurotypical participants are displayed in red, whereas data from ASD individuals are displayed in green (height threshold *p* < 0.001). Overlaps between red and green regions are displayed in yellow. Additional overlaps were seen in the fusiform cortex at lower threshold only (see main text). Activations are overlaid on an inflated brain surface. Three views are depicted: lateral view of left and right hemispheres (left and right side of the figure, respectively) and ventral view (center of the figure). Note that in the ventral view the right hemisphere is left to the left hemisphere. *FFA*, fusiform face area; *Amy*, amygdala; *pSTS*, posterior portion of the superior temporal sulcus; *mOFC*, medial orbitofrontal cortex; *r* and *l*, right and left hemisphere respectively.

*Face Localizer*. Data from the Face localizer are displayed in Table 2 and Figure 4. We tested, in each group, whether there were significant differences in neural activity between the Face and House categories. The contrast *Faces > Houses* confirmed, in both neurotypical (Figure 4, red clusters) and ASD individuals (green clusters), an involvement of the amygdala and of the posterior portion of the superior temporal sulcus in the two hemispheres. Controls also exhibited activation the medial orbitofrontal cortex. No fusiform activation was found in either group at the whole-brain threshold. We therefore performed additional region-of-interest analyses restricted to those voxels that were part of these fusiform gyrus as described by predefined anatomical masks (AAL database—Tzourio-Mazoyer et al., 2002). In Controls, we found bilateral activation of the fusiform gyrus, ~45 mm posteriorly from the anterior commissure, over and around the region usually identified as FFA. No suprathreshold activation was found in ASD participants, although at a less stringent height threshold (corresponding to *p* < 0.005 uncorrected) activation was found around the same FFA coordinates as defined in the Control group for both the right (12 consecutive voxels centered at the coordinates *x* = 42, *y* = −48, *z*= −21) and left hemisphere (three consecutive voxels centered at *x* = −39, *y* = −48, *z* = −21). The opposite contrast (*Houses > Faces*) implicated large portions of the parahippocampal gyrus, extending to the calcarine cortex and the medial portion of the middle occipital gyrus, in both groups similarly. When testing for the interaction between the grouping factor and the stimuli employed (*Faces* vs. *Houses)*, we found no suprathreshold effects.

**Table 2:** **fMRI analysis: face localizer**.

	**Side**	**Coordinates**			***t***_**(18)**_	**Cluster size**
		***x***	***y***	***z***		
**CONTROLS: FACES > HOUSES**						
Post. superior temporal sulcus	R	60	−60	15	6.43	226^†^
	L	−51	−69	21	4.97	66^*^
Amygdala	R	21	−6	−21	6.81	103^‡^
	L	−15	−9	−21	8.65	161^†^
Medial orbitofrontal cortex	M	3	48	−12	6.11	183^†^
Fusiform gyrus (FFA)	R	42	−48	−21	6.71^1^	8
	L	−39	−45	−29	5.62^1^	6
**ASD: FACES > HOUSES**						
Post. superior temporal sulcus	R	57	−72	12	5.78	218^†^
	L	−54	−60	18	7.90	255^†^
Amygdala	R	18	−3	−21	6.38	152^‡^
	L	−15	−9	−21	6.65	207^†^
**CONTROLS: HOUSES > FACES**						
Middle occipital gyrus	R	33	−87	18	7.01	2501^†^
	L	−30	−90	21	9.95	
Calcarine sulcus	R	21	−84	−12	7.11	
	L	−18	−78	−15	8.79	
Parahippocampal gyrus	R	24	−45	−15	8.09	
	L	−27	−39	−18	7.87	
**ASD: HOUSES > FACES**						
Middle occipital gyrus	R	33	−87	18	7.59	1815^†^
	L	−30	−93	18	10.21	
Calcarine sulcus	R	27	−87	−12	4.75	
Parahippocampal gyrus	R	30	−45	−9	7.85	
Calcarine sulcus	L	−27	−84	−12	7.38	390^†^
Parahippocampal gyrus	L	−21	−54	−12	9.13	

*Regions showing significant activations associated with the 1-back task in the Face localizer session. Coordinates (in standard MNI space) refer to maximally activated foci: x = distance (mm) to the right (+) or the left (−) of the midsagittal line; y = distance anterior (+) or posterior (−) to the vertical plane through the anterior commissure (AC); z = distance above (+) or below (−) the inter-commissural (AC-PC) line. L and R refer to the left and right hemisphere, respectively. M refers to medial clusters*.

† p < 0.001;

‡ p < 0.01;

* *p < 0.05 corrected at the cluster level for the whole brain (underlying height threshold: p < 0.001, uncorrected)*.

1 *p < 0.05 corrected at the voxel level for the fusiform gyrus bilaterally*.

In sum, data from this localizer session successfully identified neural structures most sensitive to face stimuli, indicating the recruitment of similar portions of the fusiform cortex and amygdala in each group (although the evidence of FFA activity in ASD individuals was obtained with a more liberal threshold).

As the functional localizer aimed at mapping in our population those portions in fusiform cortex and amygdala that were most sensitive to full-band face stimuli in each group, we then used the results of the localizer session to create a mask which could serve as region of interest in all subsequent analyses. This mask was built following anatomical and functional criteria, as it included voxels which (1) were part of either the fusiform gyrus or the amygdala according to predefined anatomical masks (AAL database) and (2) exhibited significant [*t*_(18)_ > 1.73, *p* < 0.05 uncorrected] increase of neutral activity for faces (as opposed to houses) in each group [conjunction ((*Faces* > *Houses*)_Controls_ ∩ (*Faces > Houses*)_ASD_)]. The resulting mask, which was smoothed (8 mm FWHM Gaussian kernel) and subsequently re-binarized to minimize spatial inhomogeneities, encompasses that part of the fusiform-amygdala face network that is common to both groups.

#### Effects of LSF emotional expressions

**Figure 5:**
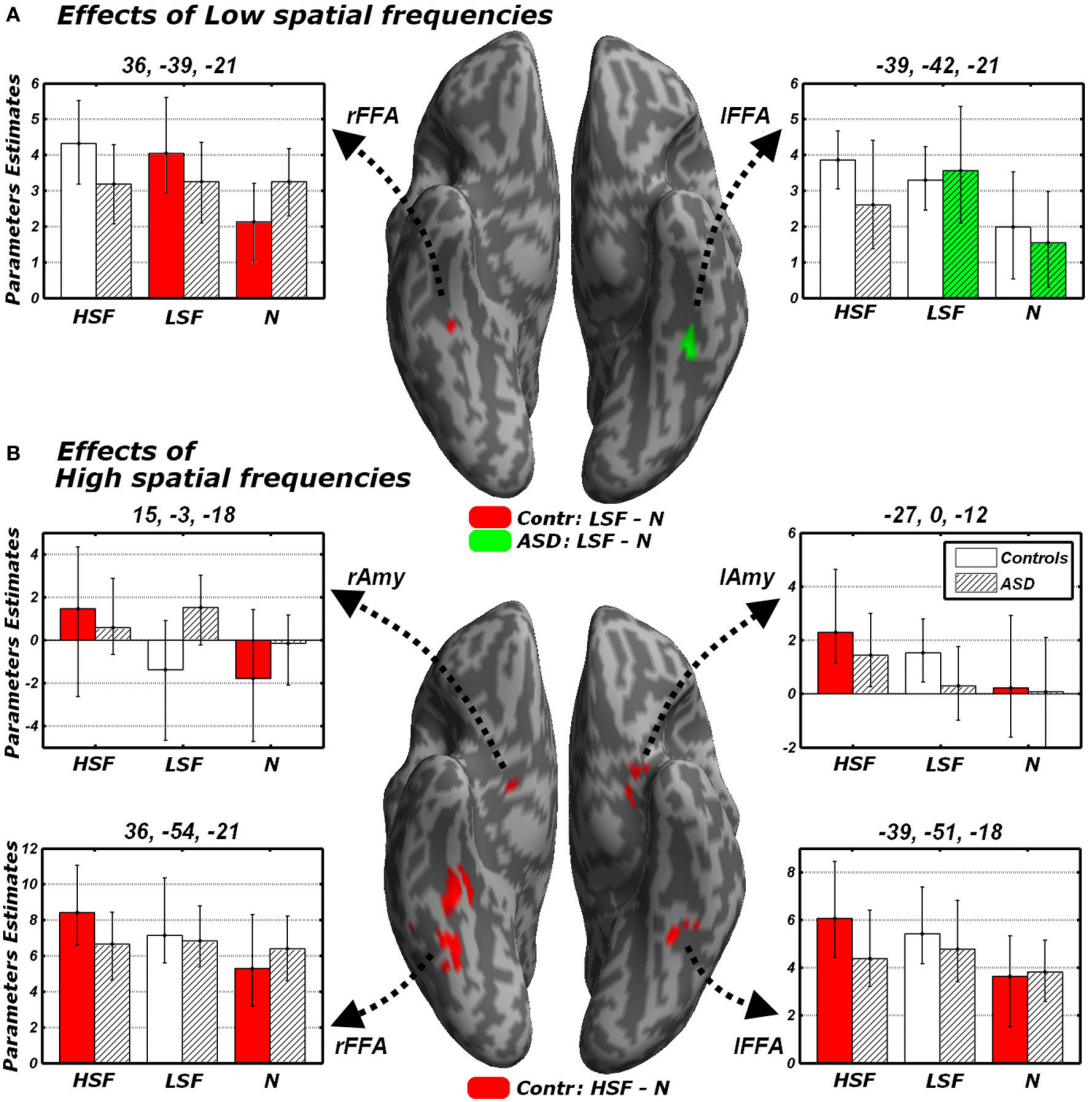
**Discrimination task: independent effects of emotional information in either LSF (A) or HSF (B) relative to neutral control stimuli**. Whole-brain maps showing significant increase of neural activity associated with **(A)** the contrast *LSF − N* or **(B)** the contrast *HSF − N* in Controls (red blobs) and ASD individuals (green blobs). Activations are overlaid on an inflated brain surface. Suprathreshold activations were found in fusiform gyrus (over and around FFA) and the amygdala. Parameters extracted from each cluster are displayed bootstrap-based 95% confidence intervals of the mean. Empty bars refer to data from Controls whereas striped bars refer to data from ASD individuals. Color codes on the bar graphs refer to conditions in the statistical test used to identify each region. *FFA*, fusiform face area; *Amy*, amygdala; *r* and *l*, right and left hemisphere respectively.

We focused on that portion of the data in which Controls carried out the gender discrimination task and tested for increases of neural activity associated with LSF expressions, relative to neutral stimuli [(*LFL* + *LFH* + *LHL* + *LHH*)/2 − (*NL* + *NL*), hereafter *LSF* - *N*]. When correcting for multiple comparisons across the whole brain we found no suprathreshold activation. However, when applying small-volume correction on those portions of the fusiform gyrus and amygdala identified in the localizer (see above), we found a significant increase of neural activity in the right fusiform cortex (see Table 3 and Figure 5A, red blob). This right fusiform activation was close, not only to the location previously identified by Winston et al. (2003b) in the same contrast (distance between the local maxima from the two studies ~11 mm), but also to the right FFA cluster isolated in the same group during the face localizer and displayed in Figure 4 (local maxima distance ~15 mm). No suprathreshold effect was found in the amygdala (similar to Winston et al., 2003b, but see Vuilleumier et al., 2003 who used simple band pass filtered stimuli).

**Table 3:** **fMRI analysis: effects of HSF and LSF emotional cues**.

	**Side**	**Coordinates**			***t***_**(250)**_	**Cluster size**
		***x***	***y***	***z***		
**CONTROLS: LSF > N** (LFL + LFH + LHL + LHH)/2 − (NL + NH)						
Fusiform gyrus (FFA)	R	36	−39	−21	3.58^1^	1
**ASD: LSF > N** (LFL + LFH + LHL + LHH)/2 − (NL + NH)						
Postcentral gyrus	L	−48	−21	30	4.99	241^†^
Supramarginal gyrus	L	−60	−33	30	4.65	
Postcentral gyrus	R	57	−9	39	4.53	78^*^
Fusiform gyrus (FFA)	L	−39	−42	−21	4.23^1^	15
**CONTROLS: HSF > N** (HFL + HFH + HHL + HHH)/2 − (NL + NH)						
Fusiform gyrus (FFA)	R	36	−54	−21	4.59	68^*^
	L	−39	−51	−18	5.15^1^	13
Amygdala	R	15	−3	−18	3.67^1^	2
	L	−27	0	−12	4.14^1^	12
**CONTROLS: HSF > LSF** [(HFL + HFH + HHL + HHH) − (LFL + LFH + LHL + LHH)]						
Angular gyrus	R	45	−57	27	3.76	576^†^
Precuneus	R	12	−54	9	4.94	
Calcarine gyrus	R	9	−69	18	4.30	
Post. cingulate cortex	L	−9	−48	33	4.30	
Precuneus	L	−12	−54	15	4.79	
Medial orbitofrontal cortex	R	6	57	−3	4.54	561^†^
Caudate nucleus	R	6	12	−3	5.27	
	L	−15	18	−3	4.48	
Middle occipital gyrus	L	−21	−93	6	4.95	256^†^
Calcarine gyrus	L	−6	−96	−6	4.80	
Fusiform gyrus (post. part)	R	30	−69	−3	4.38	150^‡^
Lingual gyrus	R	9	−75	−3	3.90	
Amygdala	R	18	−3	−18	4.26^1^	10
**GROUP-INTERACTION: (HSF > LSF)_**Controls**_ > (HSF > LSF)_**ASD**_**						
Calcarine gyrus	M	−3	−96	0	5.42	435^†^
Lingual gyrus	R	12	−75	−3	4.66	
Lingual gyrus	L	−15	−84	0	4.03	
Caudate nucleus	R	18	18	0	4.83	100^‡^
Lateral occipital cortex	L	−48	−69	−15	4.38	100^‡^
Fusiform gyrus (FFA)	L	−39	−45	−18	3.65^1^	3
Amygdala	R	18	−3	−21	4.26^1^	11

*Regions showing significant activation associated with the discrimination task*.

† p < 0.001;

‡ p < 0.01;

* *p < 0.05 corrected at the cluster level for the whole brain (underlying height threshold: p < 0.001, uncorrected)*.

1 *p < 0.05 corrected at the voxel level for FFA and amygdala bilaterally as described by the localizer data*.

One of the key questions of the present study was to assess whether this increase of neural activity in FFA for LSFs (as found in Controls) was absent or preserved in ASD individuals. We therefore examined the sessions in which ASD participants performed the gender discrimination task and tested for the same contrast *LSF − N*: this revealed an activation of the left FFA, in a location very symmetrical to that identified in Controls (see Figure 5A, green blob—local maxima distance between this cluster and the left FFA cluster identified in the same group ~6 mm). No effect was found in the right fusiform gyrus or in the amygdala even at the most liberal thresholds.

We further explored putative group differences in the neural response to LSF emotional expressions by testing the interaction between the contrast *LSF − N* and the grouping factor. In particular, we tested for regions in which the differential activity between LSF and neutral expressions in Controls was not only larger than 0 (as already tested above), but also larger than the same differential activity in ASD [i.e., (*LSF* − *N*)_Controls_ − (*LSF* − *N*)_ASD_]. However, as this test also isolates regions with no difference between LSF and neutral expressions in Controls, but with reduced activity for LSF expressions (as opposed to N) in ASD individuals, we excluded from our search those regions that were implicated (*p* < 0.05 uncorrected) in the contrast *N* − *LSF* in ASD (exclusive masking). This test revealed no differential effect, neither when correcting for multiple comparisons for the whole brain, nor when focusing on the face-sensitive portions of fusiform gyrus/amygdala. With a similar logic, we tested for regions in which the differential activity between LSF and neutral expressions was larger in ASD individuals than in Controls [i.e., (*LSF* − *N*)_ASD_ − (*LSF* − *N*)_Controls_]. Also this test led to no suprathreshold effects, including for the fusiform gyrus/amygdala at liberal thresholds.

In sum, not only we found reliable evidence in the neurotypical brain for a role of LSF inputs conveying emotional expression information to FFA (Winston et al., 2003b), but we also found equivalent (although contralateral) effects in ASD, suggesting that these LSF inputs are preserved in these participants. Furthermore, direct comparison of the effects identified in each group failed to reveal any significant difference.

#### Effects of HSF emotional expressions

We next tested for regions exhibiting suprathreshold activity when emotional face expressions were conveyed by HSFs. We first computed, in Controls, the contrast [(*HFL+HFH + HHL+HHH*)/2 − (*NL + NL*), hereafter *HSF* − *N*] which revealed enhanced bilateral activity in the fusiform gyrus, over and around FFA, as well as in the Amygdala (see Table 3 and Figure 5B, red blobs). Critically, the fusiform clusters were proximal to the FFA clusters delineated with the functional localizer in the same group (Figure 4) and to the clusters identified by the main effect of LSF expressions (Figure 5A, red blobs). No effect was found for the contrast *HSF* − *N* when ASD participants carried the discrimination task.

We then tested directly whether the differential activity observed in Controls was larger, not only than 0, but also of its homologous in ASD individuals *via* an interaction test [(*HSF* − *N*)_Controls_ − (*HSF* − *N*)_ASD_, excluding voxels sensitive to (*N* − *HSF*)_ASD_]. We found no suprathreshold activity, neither when correcting for the whole brain, nor when applying a small volume correction. It should be mentioned, however, that under an uncorrected extent threshold (underlying height threshold of *p* < 0.001), we found five consecutive voxels on right FFA [local maxima: *x* = 33, *y* = −51, *z* = −21, *t*_(162)_ = 3.57], proximal to the cluster previously implicated when testing effects of HSF expressions (local maxima distance <5 mm). No region exhibited HSF increases of activity specific for ASD individuals [(*HSF* −*N*)_ASD_ − (*HSF* − *N*)_Controls_], neither when correcting for the whole brain, nor when inspecting the fusiform gyrus and the amygdala with more liberal approaches.

In sum, the analysis of HSF effects during the discrimination task revealed significant increases of neural activity in FFA and amygdala to emotional expressions in the Controls exclusively. For the right FFA, such increase was not only larger than 0, but also larger than the homologous (non-significant) effect measured in ASD individuals.

#### Direct comparisons between LSF and HSF expressions

We also compared differential responses to LSF or HSF expressions, not against the control neutral condition, but against each other. Unlike the analysis conducted insofar—which identified regions sensitive to one frequency band, irrespective of their sensitivity also to the other bands—these direct comparisons now probed for any region that would code *preferentially* for emotional information conveyed by specific frequencies.

**Figure 6:**
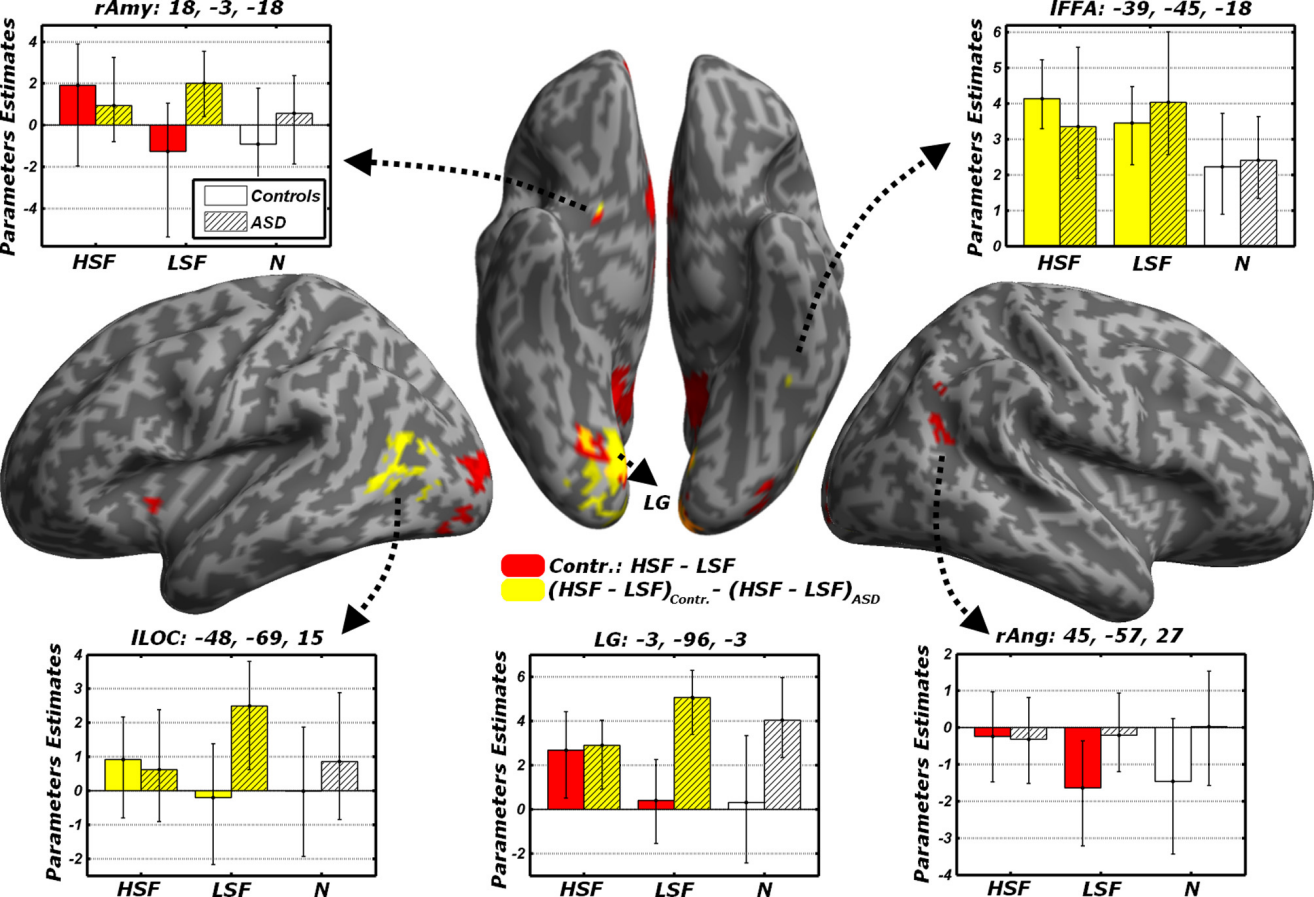
**Discrimination task: dissociated responses to LSF or HSF emotional information**. Whole-brain maps showing significant increase of neural activity to HSF but not LSF emotional cues in the contrast *HSF* − *LSF* for Controls (red blobs). Furthermore, regions significantly associated with the interaction contrast (*HSF* − *LSF*)_Controls_ − (*HSF* − *LSF*)_ASD_, which tests group-specific differential responses to HSF and LSF emotional cues are also displayed (yellow blobs). Data are overlaid on an inflated brain surface. Parameters extracted from each cluster are displayed, with bootstrap-based 95% confidence intervals of the mean. Empty bars refer to data from Controls, whereas striped bars refer to data from ASD individuals. Color codes on the bar graphs refer to conditions in the statistical test used to identify each region. *Amy*, amygdala; *FFA*, Fusiform Face Area; *LOC*, lateral occipital cortex; *LG*, lingual gyrus; *Ang*, angular gyrus; *r* and *l*, right and left hemisphere respectively.

When testing for differential responses to LSF expressions [contrast (*LFL* + *LFH* + *LHL* + *LHH*) – (*HFL* + *HFH* + *HHL* + *HHL*), hereafter *LSF* − *HSF*], we found no suprathreshold effect in neither in Controls, nor in ASD individuals. We then tested the converse contrast *HSF* − *LSF*, which probed for any region processing emotional facial expressions from HSF cues preferentially to LSF cues. For Controls, this contrast elicited large activations within the ventral occipital cortex, including the posterior portions of the fusiform gyrus. Further activations were found in the right angular gyrus, the precuneus/posterior cingulate cortex, the ventral striatum bilaterally, the medial orbitofrontal cortex and, when applying small volume correction, the right amygdala (see Table 3 and Figure 6, red blobs). No effects (even at the most liberal thresholds) were observed when the same contrast was run on the ASD group.

In sum, these data confirm in Controls the recruitment of a widespread network processing emotional face expression from HSFs preferentially to LSFs. Such network was not reported in ASD individuals, not even at the most liberal thresholds. It is therefore possible that the same regions processing preferentially HSF in Controls might exhibit different sensitivity to spatial frequency emotional cues in ASD. We formally tested this via a cross-over interaction contrast (*HSF* − *LSF*)_Controls_ − (*HSF* − *LSF*)_ASD_, comparing the differential sensitivity between HSF and LSF emotional cues across groups. As fully described in Figure 6 (yellow blobs) and Table 3, this analysis confirmed the role played by the lingual gyrus, the ventral striatum and the right amygdala. Furthermore, this analysis also implicated the left lateral occipital cortex and left FFA, thus confirming how this region seems more sensitive to HSF expressions in Controls and, concurrently, to LSF expressions in ASD individuals (see also Figure 5).

#### Effects of the reported gender and of emotional valence

**Figure 7:**
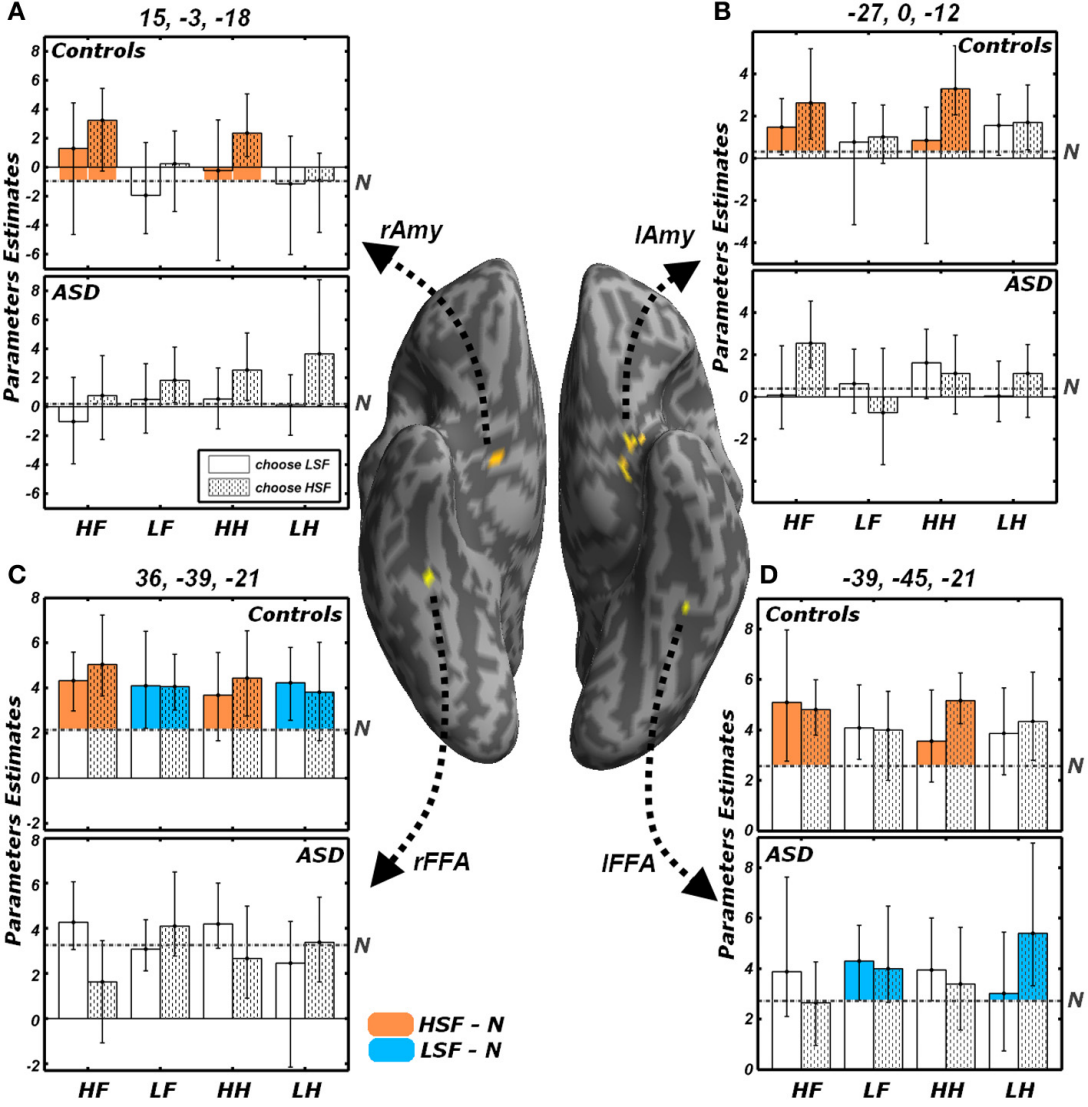
**Effects of the reported gender and of emotional valence**. Average parameters extracted from representative voxels of **(A)** right Amygdala, **(B)** left Amygdala, **(C)** right FFA and **(D)** left FFA. The left and right FFA voxels were chosen as exhibiting significant conjoint activity for the contrasts *LSF − N* (as shown in Figure 5A) and *HSF* − *N* (Figure 5B). Amygdalar voxels were those composing the clusters depicted in Figure 5B. The four regions are displayed in yellow on a ventral portion of an inflated human brain. For each of these four regions, average parameters estimates are displayed with bootstrap-based 95% confidence intervals of the mean. Data from different groups are displayed in separate subplots. Empty bars refer to trials in which participants choose the gender depicted by the LSF, whereas dotted bars refer to trials in which the HSF gender was chosen. The average activity associated with the neutral condition is displayed as a gray horizontal dash-dotted line. The portions of the bars which exceed the activity of the neutral condition are colored according to the functional test with which the regions were defined. Regions isolated through the contrast *LSF* − *N* (Figure 5A) display the bars associated with LSF conditions colored in blue; instead regions isolated through the contrast *HSF* − *N* (Figure 5B) display the bars colored in orange. *HF*, HSF fearful expression; *LF*, LSF fearful expression; *HH*, HSF happy expression; *LH*, LSF happy expression; *N*, Neutral expression; *Amy*, amygdala; *FFA*, Fusiform Face Area; *r* and *l*, right and left hemisphere respectively.

All analyses conducted insofar were run regardless of the behavioral performance and of the emotional valence. Figure 7 illustrates the activity parameters extracted from those FFA and Amygdalar voxels identified by the contrasts *LSF* − *N* and *HSF* − *N* (Figure 5). Visual inspection of these data suggests how in some cases the differential activity between emotional and neutral expressions described above might be biased by the task demands. In particular, the amygdala exhibited, in Controls, a differential increase in activity for HSF expressions; however, further in-depth analyses on the extracted parameters revealed a general marginal preference for all trials in which HSF were “preferred” for the gender discrimination [choose HSF vs. choose LSF: right Amygdala *t*_(9)_ = 2.04, *p* = 0.072; left Amygdala *t*_(9)_ = 2.05, *p* = 0.071]. Instead, amygdala activity seemed unaffected by the kind of emotion displayed by HSFs [fearful vs. happy: right Amygdala *t*_(9)_ = 0.87, n.s.; left Amygdala *t*_(9)_ = −0.13, n.s.]. Keep in mind that the contrast *HSF* − *N*, implicating the amygdala in our earlier analyses (Figure 5B), was calculated by weighting equally the two possible gender choices.

On the other hand, we found that FFA activity to HSF (in Controls) and to LSF (in Controls for the right hemisphere, and ASD individuals in the left hemisphere) was globally unaffected by participants' behavior or by emotional valence [|*t*|_(9)_ always <1.60]. Visual inspection of Figure 7D, suggests that, in ASD individuals, the processing of LSF happy expressions might elicit larger activity in left FFA for those trials in which a HSF gender was chosen as opposed to a LSF gender. This was confirmed by an *ad-hoc* comparison [LHH vs. LHL, *t*_(9)_ = 2.76, *p* < 0.05].

Finally, we extended the results obtained in FFA and amygdala to the whole brain, by assessing for each group the putative effects of the behavioral choice (HSF vs. LSF gender) and of emotional valence (fearful vs. happy). However, this analysis led, in its most relevant contrasts, to no suprathreshold activity. Specifically, neither Controls nor ASD individuals exhibited any suprathreshold effect associated with emotional valence, neither when testing the overall main effect [contrast (*LFL* + *LFH* + *HFL* + *HFH*) − (*LHL* + *LHH* + *HHL* + *HHH*) and inverse], nor when focusing only on those trials in which emotions were conveyed by specific frequency bands [contrasts (*LFL* + *LFH*) − (*LHL* + *LHH*), (*HFL* + *LFH*) − (*HHL* + *HHH*), and inverses]. No suprathreshold effect was found when testing whether there were regions affected by participants' choice [contrast (*LFL* + *LHL* + *HFL* + *HHL*) − (*LFH* + *LHH* + *HFH* + *HHH*) and vice versa]. As in the case of the amygdala (Figures 7A,B), we tested putative effects of choice within those frequency bands conveying emotional information (choose HSF > choose LSF only for HSF emotional expressions, or choose LSF > choose HSF only for LSF expressions), but no significant effect was found in any of the groups. No suprathreshold effect was found associated with the interaction between the frequency conveying an emotional expression and the frequency promoting the gender choice, specifically when searching for regions with higher activity in trials in which the face with an emotional expression was chosen rather than neglected [contrast (*LFL* + *LHL* + *HFH* + *HHH*) − (*LFH* + *LHH* + *HFH* + *HHH*)]. Finally, in keeping with our behavioral finding that participants' response was affected by the emotional content of happy expressions only, we excluded from the interaction contrast those trials displaying fearful faces [contrast (*LHL* + *HHH*) − (*LHH* + *HHH*)], but even in this case we found no suprathreshold activity.

#### Passive viewing trials

**Figure 8:**
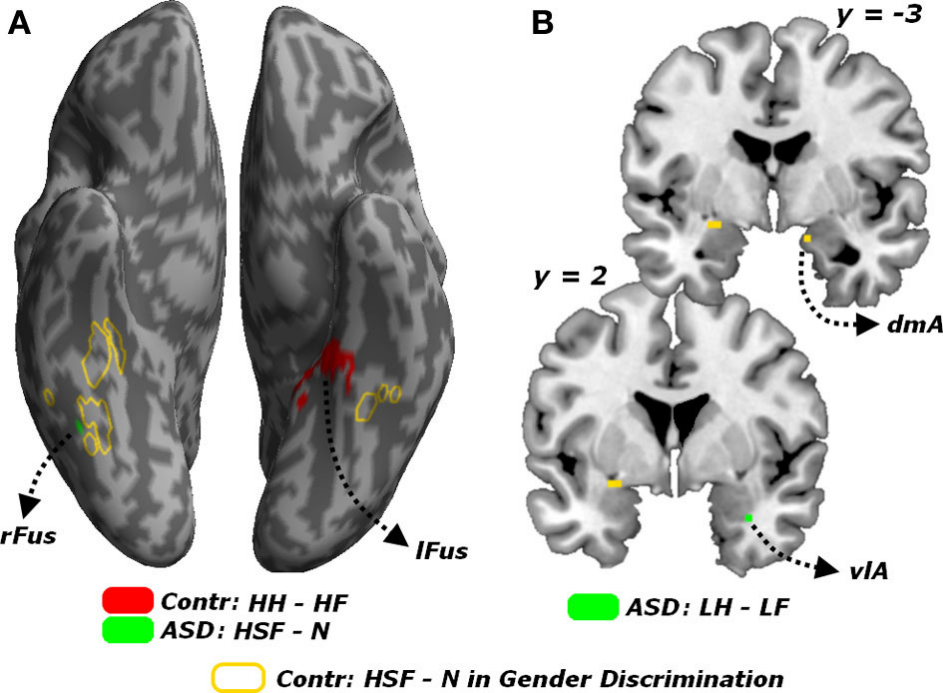
**Passive viewing sessions. (A)** Whole-brain maps showing significant increase of neural activity associated with the contrast *HH* − *HF* in Controls (red blob) the contrast *HSF - N* in ASD individuals (green blob). Activations are overlaid on an inflated brain surface. **(B)** Coronal sections (*y* = −3, 2) displaying the increase of neural activity for the contrast *LH* − *LF* in ASD individuals (green blobs) in the right amygdala. For both **(A,B)** portions of fusiform and amygdalar cortex implicated in the contrast *HSF* − *N* in earlier analysis on the gender discrimination sessions are displayed in yellow. *Fus*, Fusiform Gyrus; *r* and *l*, right and left hemisphere respectively. *dmA* and *vlA*, dorsomedial and ventrolateral portions of the amygdala.

Finally, all effects associated with the passive viewing trials are displayed in Table 4 and can be summarized as follows. No region was uniquely recruited by the perception of LSF emotional expressions as opposed to neutral stimuli (*LF* + *LH*)/2 − *N*, neither in Controls nor in ASD individuals. Instead, ASD individuals (but not Controls) exhibited increased activity in the right fusiform gyrus for HSF expressions [contrast (*HF* + *HH*)/2 − *N*], in proximity to the region identified through the same contrast in Controls when testing the gender discrimination sessions (see Figure 8A, green blob). We then inspected any effect of emotional valence, both as a global main effect [contrast (*LF* + *HF*) − (*LH* + *HH*) and inverse] and by analyzing separately each frequency band. Controls exhibited only enhanced activity of the most anterior portion of the left fusiform gyrus, extending to the parahippocampal cortex, for HSF happy (relative to HSF fearful) expressions (see Figure 8A, red blobs).

**Table 4:** **fMRI analysis: regions showing significant activation associated with the passive viewing sessions**.

	**Side**	**Coordinates**			***t***_**(250)**_	**Cluster size**
		***x***	***y***	***z***		
**ASD: HSF > N** (HF + HH)/2 − N						
Fusiform gyrus (FFA)	R	42	−54	−15	4.08^1^	3
**CONTR: HSF_HAPPY > HSF_FEARFUL** HH − HF						
Fusiform gyrus	L	−27	−36	−18	4.58	81^*^
Parahippocampal gyrus	L	−15	−39	−6	3.67	
**ASD: FEAR > HAPPY** (LF + HF) − (LH + HH)						
Anterior insula	L	−27	27	12	4.09	66^*^
**ASD: LSF_HAPPY > LSF_FEARFUL** HF − HH						
Amygdala	R	33	3	−27	3.62^1^	1
**ASD: HSF_HAPPY > HSF_FEARFUL** HH − HF						
Temporo−parietal−junction	R	45	−63	33	4.35	104^‡^
	L	−45	−66	27	5.20	307^†^
Superior frontal sulcus	L	−24	21	45	5.69	122^‡^
Posterior cingulate cortex	M	−6	−57	48	5.05	156^‡^

† p < 0.001;

‡ p < 0.01;

* *p < 0.05 corrected at the cluster level for the whole brain (underlying height threshold: p < 0.001, uncorrected)*.

1 *p < 0.05 corrected at the voxel level for FFA and amygdala bilaterally as described by the localizer data*.

On the other hand, ASD individuals exhibited increased activity in the left middle-anterior insula for fearful (as opposed to happy) expressions, regardless of the spatial frequency. Such effect was not observed when repeating the analysis separately for each frequency band. Furthermore, in ASD individuals, LSF happy expressions triggered (compared to LSF fearful expressions) enhanced activity in the most ventro-lateral part of the right amygdala (Figure 8B, green blob). Finally, in ASD individuals, the contrast (*HH* − *HF*) elicited significant differential activation in the temporo-parietal junction (bilaterally), the posterior cingulate cortex and the left superior frontal sulcus.

In sum, in sharp contrast with the case of the Gender Discrimination task, during the passive viewing sessions ASD individuals exhibited increased neural responses in portions of the core face network for emotional facial expressions, including those conveyed by HSFs.

## Discussion

We tested for the independent contribution of HSF or LSF visual inputs to brain regions critical for face processing, by engaging individuals with ASD and matched neurotypical Controls in a gender discrimination task with hybrid face stimuli. We found that, compared to Controls, individuals with ASD exhibited a reduced sensitivity to emotional information conveyed by the cortical HSF pathway, but were as sensitive as Controls to information conveyed by the LSF pathway. This was observed both in the portion of fusiform gyrus sensitive to face stimuli (FFA), when measuring the neural responses to emotional expressions in either frequency against control neutral faces (Figure 5), and in both the ventral occipital cortex and the amygdala when testing HSFs against LSFs (Figure 6, red blobs). FFA, the ventral occipital cortex and the amygdala were also showed a significant interaction reflecting that the increased activity for HSF expressions observed in Controls was reliably larger than this effect in ASD individuals (Figure 6, yellow blobs). Furthermore, both FFA and amygdala responses to emotional cues seem independent from the emotional valence, whereas they were modulated by the participants' choices in the gender task—at least for the amygdala (Figure 7). Critically, these data cannot be interpreted as ASD being characterized by a generalized insensitivity to HSF cues conveyed by the cortical pathway *per se*, because posterior visual cortical regions responded to HSF emotional information in ASD individuals during the passive viewing sessions (Figure 8). Instead, the data suggest decreased sensitivity to HSF information when processing global facial features, such as during active gender discrimination.

### Low- and high-frequency processing in ASD

The hybrid nature of our stimuli, and LSF and HSF cut-offs adopted in keeping with our previous studies (<6 c/fw and >24, c/fw respectively—Vuilleumier et al., 2003; Winston et al., 2003b; Pourtois et al., 2005) served two main purposes: first, they allowed co-occurrent, and yet dissociable, recruitment of parvocellular and magnocellular pathways; second they insured that spatial frequency information conveyed by each pathway provided coarse (LSF) and fine-grain (HSF) facial cues that were equally distant from optimality. With this set of stimuli, we found no behavioral difference between ASD individuals and Controls. Group differences were observed only when measuring neural responses, specifically for fine-grained information that is uniquely conveyed by the cortical pathway. Earlier studies using gratings stimuli of LSFs or HSFs found comparable contrast thresholds in ASD individuals and Controls (Bertone et al., 2005; De Jonge et al., 2007; but see Davis et al., 2006, for differences in HSF), but nevertheless documented atypical neural responses in ASD (for LSF, Boeschoten et al., 2007b; Vlamings et al., 2010; for HSF Boeschoten et al., 2007b; Milne et al., 2009).

Faces are much more complex stimuli as they are processed through the integration of co-occurrent HSF and LSF information arising from each pathway. Notably, earlier studies using simple band-pass filtered or hybrids faces often reported that ASD individuals might be more biased in favor of HSF than LSF (Deruelle et al., 2004, 2008), and exhibit atypical neural responses to LSF faces (Vlamings et al., 2010). It should be mentioned, however, that these studies differed from ours in many aspects, such as the recruitment of children (see Rondan and Deruelle, 2004, for a lack of effects in adults), the task employed (see Deruelle et al., 2008, who reported no HSF biases in gender discrimination task) and, critically, the use of a more liberal LSF cutoff (<12 c/fw). Indeed, psychophysical investigations in neurotypical individuals have consistently described that face information is optimally processed from intermediate frequency bands (between 8-16 c/fw – Costen et al., 1994, 1996; Gold et al., 1999; Näsänen, 1999; Parker and Costen, 1999; Boutet et al., 2003; Collin et al., 2006; Watier et al., 2010). In this perspective, previous studies should not be interpreted as showing atypical processing of LSFs *per se*, but of those intermediate frequencies optimal for face processing. Consistently with this conjecture, a study employing face stimuli filtered under a more stringent LSF cutoff (< 5 c/fw—thus, outside the range 8–16 c/fw) reported no difference in neural activity between ASD children and matched Controls (Boeschoten et al., 2007a).

### Gender discrimination in ASD

The gender discrimination task employed here served the purposes of our study in three critical aspects. First, this task chiefly requires the inspection of faces from a global point of view, as shown by decreased performance when the face stimuli are inverted, scrambled, or when the upper and lower halves are misaligned (Zhao and Hayward, 2010). Second, the gender of hybrid stimuli can be discriminated by relying on either LSF or HSF bands (equally from both bands in Schyns and Oliva, 1999; slightly LSF-biased in Winston et al., 2003b). Third, the discrimination is influenced by the (task-irrelevant) emotional expressions of one of the two faces composing the hybrid, as shown by our behavioral data: happy expressions bias the judgment toward the frequency bands in which these are conveyed (see Figure 3), suggesting that gender discrimination itself might actually be combined with a parallel and automatic extraction of the emotional information from the face, including its valence (Vuilleumier, 2007; Vuilleumier and Righart, 2011). In Controls, the increase of activity in the fusiform gyrus when either frequency band conveyed an emotional expression might be a neural signature of such extraction.

In the ASD group, no increase of neural activity was associated with HSF emotional expressions during active gender discrimination, suggesting lower use of emotional information from HSF in this condition, or alternatively increased efficiency at ignoring task-irrelevant information from one specific frequency band. In this perspective, one might expect ASD individuals to be conversely more biased toward LSFs than Controls in their judgments on hybrid faces, a pattern also suggested by visual inspection of behavioral data in Figure 3. Unfortunately, group differences in these behavioral results did not reach statistical significance. In any case, the reported differences in brain activation cannot merely be explained by performance, as LSF- and HSF-biased trials were modeled independently in each participant, and both weighted equally on all subsequent analytical stages regardless of individual idiosyncratic response-biases. We are therefore confident that our results truly reflect differences in visual perceptual processing.

### Global and local processing in ASD

At a first sight, ASD's decreased sensitivity to high-frequency information (only during the discrimination task) might be considered at odds with a large body of evidence suggesting how ASD processing of visual stimuli might be biased in favor of detailed (fine-grain) information, at the expense of the global picture. Indeed, ASD individuals have been reported to be more proficient than Controls in tasks in which the global information conflicts with locally-displayed targets (Shah and Frith, 1983; Happé, 1996; Pellicano et al., 2005; Simmons et al., 2009; Almeida et al., 2013) but, at the same time, less proficient in detecting coherent global patterns when intermingled with distracting local information (Spencer et al., 2000; Milne et al., 2002; Pellicano et al., 2005; Spencer and O'Brien, 2006; Tsermentseli et al., 2008).

Please note, however, that the distinction between local vs. global information from earlier studies is not necessarily equivalent to a distinction between HSF vs. LSF information. Indeed, whereas local information is indubitably conveyed by HSF, global information can, at least in principle, be obtained by all frequency ranges, with some critical differences: on the one side, LSF provides global cues from visual stimuli (e.g., a face) *regardless* of local information, instead HSF can provide global cues by integrating multiple local details together. In this perspective, our findings of decreased HSF-related activity in ASD can be reconciled with earlier accounts only under the assumption that ASD local biases are reflective of a difficulty in seeing the whole through the integration of many details. Consistently with this assumption, recent studies investigated visual integration by using two independent kinds of stimuli: (1) stimuli whose global properties are retained regardless of the details (hierarchical figures, Navon, 1977), for which ASD individuals perform comparably to Controls Deruelle et al., 2006; Rondan and Deruelle, 2007; (2) stimuli whose global properties are retained only from the combined information of many local features (e.g., gestalt illusions of similarity, proximity, etc.), for which ASD individuals exhibit difficulties relative to Controls (Brosnan et al., 2004; Deruelle et al., 2006; Bölte et al., 2007; Rondan and Deruelle, 2007). Please note that in the former kind of stimuli, the global information was available at a coarse level of resolution, thus retainable even after low-pass spatial filtering. This is not necessarily the case for the latter kind of stimuli, in which the global information may also be obtained from information at a more fine-grain level (see also Dakin and Frith, 2005; Simmons et al., 2009 for similar arguments in contour integration tasks).

### Fusiform and amygdala function in ASD

Although many behavioral studies failed at documenting differences in face processing between ASD individuals and Controls, more systematic effects were reported by fMRI studies including reduced neural responses in the fusiform gyrus and the amygdala when processing (emotional or neutral) facial expressions (Baron-Cohen et al., 2000; Critchley et al., 2000; Schultz et al., 2000; Pierce et al., 2001; Hall et al., 2003; Hubl et al., 2003; Wang et al., 2004; Grelotti et al., 2005; Ashwin et al., 2007; Scherf et al., 2010). These results were first interpreted as ASD being characterized by an atypical development of the fusiform gyrus and/or the amygdala (e.g., Baron-Cohen et al., 2000; Schultz, 2005). However, as for other accounts that attempt to describe ASD symptomatology with the dysfunction of specific brain regions (e.g., the broken mirror hypothesis, Hamilton, 2013), these anatomical models are subjected to several critiques. First, some processes associated with the incriminated regions are often spared in ASD individuals (e.g., amygdala dysfunction should also impair emotional arousal, aversive conditioning, or reward contingency learning, but these impairments were not consistently found across studies testing ASD individuals; Gaigg, 2012; see also Zalla and Sperduti, 2013). Second, lesions in incriminated regions, even when occurring at early stages of life, do not lead to the same symptomatology of ASD (Amaral et al., 2003; Paul et al., 2010, but see Bachevalier, 1994). Third, recent studies often report comparable functional properties in the incriminated regions between ASD individuals and neurotypical Controls, when controlling for factors such attentional load, stimuli presentation time or eye movements (Hadjikhani et al., 2004, 2007; Dalton et al., 2005; Bird et al., 2006). In this perspective, ASD might not be associated with damaged fusiform gyrus or amygdala *per se*, but with atypical recruitment/modulation of these regions by high-order top-down control or attentional processes (Santos et al., 2008). Also in our ASD sample the fusiform gyrus and the amygdala did not appear to be generally impaired, e.g., due to either a regional dysfunction or a general atypicality in gazing behavior—but rather this group exhibited a selective hypoactivation for a specific class of information (HSF emotional expressions in hybrid images) and under specific task demands (gender discrimination).

Moreover, Kleinhans et al. (2008) reported decreased functional connectivity between fusiform gyrus and amygdala when ASD participants processed face stimuli, pointing to a dysfunction at the network level rather than at each of its constituent nodes. We concur with this interpretation, but also extend it by offering further insights on the nature of the dysfunction. As shown in Figure 1, the amygdala is thought to receive coarse (LSF) facial information from a direct subcortical (i.e., collicular-pulvinar) path, which may then project back to the fusiform (Winston et al., 2003b), whereas in addition the fusiform gyrus also receives fine-grained (HSF) information from a feedforward (i.e., geniculo-striate and ventral occipitotemporal) cortical path. Critically, cortical and subcortical processing of faces are integrated with each other, as shown by enhanced functional connectivity between amygdala and fusiform gyrus during face processing (Morris et al., 1998), and by the impact of amygdala damage on fusiform sensitivity to facial emotional expressions (Vuilleumier et al., 2004). Thus, within this model, we can distinguish between two independent components of the amygdala-fusiform connectivity according to the direction of the information flow. Signaling *from the amygdala to the fusiform gyrus* is supported by the modulation of fusiform responses by LSF facial information initially processed in the amygdala (see Figure 4A, but also Vuilleumier et al., 2003; Winston et al., 2003b). Conversely, signaling *from the fusiform to the amygdala* is consistent with amygdala responses being also sensitive to HSF facial information conveyed by the visual cortex (see Figure 5). Our data provide novel evidence suggesting that it is the signal in the latter (but not the former) direction that exhibits atypical properties by ASD. This in turn suggests that integrative face processing functions mediated by higher level visual cortices might be more affected by ASD than lower level subcortical pathways providing inputs to the amygdala.

### Limitations of the study and future research

Like many other neuroimaging investigations on autism, including those reviewed in this article, our dataset is penalized by the limited number of participants and by an ASD population including both individuals affected by Asperger Syndrome and High Functioning Autism (see Table 1). Low power is not necessarily detrimental for positive results, which in our case were all obtained under corrected statistical thresholds (see also Friston, 2012), but it is problematic for those tests producing null or marginal results and for which an effect could potentially still be found with a larger sample. Also the heterogeneity of the clinical sample might be an additional source of noise with detrimental effects on the power of statistical analysis. Furthermore, some of the effects might be driven by only one of the two clinical sub-groups without a possibility of further verification on corresponding subsamples. It should be stressed, however, that the distinction between Asperger Syndrome and High Functioning Autism was removed in the last edition of the Diagnostic and Statistical Manual of mental disorders (American Psychiatric Association, 2013). In this perspective, putative heterogeneities in our clinical population should be treated as any within-group variability against which the significance of effects is estimated.

In particular, low power and sample heterogeneity might account for the weak effects of valence of emotional face expressions. Indeed, participants' behavior in both groups was significantly affected by valence, while the analysis of the fMRI signal did not reveal a similar effect in the brain. We should stress, however, that this consideration is not critical for our main results, since a lack of valence effects in the fusiform gyrus and the amygdala is plausible with respect to earlier accounts (Sander et al., 2003; Surguladze et al., 2003; Winston et al., 2003a). Interestingly, however, during the passive task, ASD individuals exhibited increased activity for LSF happy as opposed to LSF fearful expressions in the right amygdala. This activation arose in a ventrolateral portion of the amygdala, whereas earlier effects associated with the discrimination task arose in a more dorsal and medial location (Figure 8B). Parcellation of the human amygdala has been carried out with both cytoarchitectonic (Amunts et al., 2005) and connectivity-based approaches (Bzdok et al., 2013), and suggest that the different effects in Figure 8B might concern different sub-regions. Future research with high-resolution fMRI techniques is needed to investigate more specifically how ASD impairments in face and emotional processing might relate to different subregions of the amygdala.

Furthermore, caution should be used to interpret group differences in their response to LSF expressions relative to the neutral control condition because, unlike for HSFs, no significant interaction with the group factor was found. We can therefore not conclude whether the lateralization displayed in Figure 5A is truly reflective of different network-organization in the two groups. Please notice that, although left FFA was identified only when testing ASD individuals, visual inspection of the parameters extracted from this region suggests that a similar effect might be present also in Controls. It is therefore plausible to assume that, like for HSFs, the greater sensitivity of Controls to LSF expressions might extend to both hemispheres.

Finally, although we are quite confident that in the gender discrimination task participants focused their attention on global aspects of the face stimuli (Zhao and Hayward, 2010), we have little control on which processes were at play during the passive viewing session, in which the only instruction was to watch the stimuli attentively. Furthermore, even if participants focused on face stimuli, we do not know whether they preferentially attended to global or local properties or shifted between both. In this perspective, the increase of neural activity observed in ASD individuals for HSF emotional information in the passive condition (Figure 8) can only be taken as evidence for spared functionality of the cortical path outside the demands of the discrimination task (see Discussion section above). Future studies will need to extend these results by using other tasks in which participants are forced to focus on local facial details, thus allowing us to determine the neural signatures associated with featural facial processing in addition to the frequency content manipulation used here.

### Conflict of interest statement

The authors declare that the research was conducted in the absence of any commercial or financial relationships that could be construed as a potential conflict of interest.
